# The academic adaptation of Southeast Asian international students in Chinese universities

**DOI:** 10.3389/fpsyg.2025.1639640

**Published:** 2025-10-09

**Authors:** Ling Shen, Ailing Luo

**Affiliations:** Chinese Language and Culture College, Huaqiao University, Xiamen, China

**Keywords:** academic adaptation, individual influencing factors, environmental influencing factors, international students from Southeast Asian to China, countermeasures

## Abstract

Employing questionnaire surveys and interviews, it has been determined that Southeast Asian students generally exhibit a moderate degree of academic adaptation. They are well - adapted in terms of instructional quality, learning capabilities, school governance, educational milieu, social interactions, and Chinese language proficiency. Factors including adaptation, nationality, student status, Chinese language competence, psychological state, learning attitudes, approaches, motivation, and abilities are of significance. Instructional quality, educational environment, school governance, and social relationships also play a role in the academic adaptation of international students. Consequently, the academic adaptation of international students can be enhanced by offering specialized psychological counseling services, establishing peer support systems, strictly implementing language proficiency evaluations, upgrading teaching facilities, and strengthening the management of international students.

## 1 Introduction

More than a decade has passed since the “Belt and Road” initiative was proposed, and China has emerged as a top destination for international students seeking higher education abroad. The number of students enrolled in mainland universities continues to rise annually. According to the Education Statistics for 2022 released on the official website of the Ministry of Education, the higher education sector welcomed 125,858 international students, including 32,443 degree-seeking students. Despite the language and cultural differences, international students must continuously adapt to their studies, daily life, as well as the teaching and management approaches of their institutions. They strive to achieve a dynamic equilibrium between their personal and academic environments until they complete their education. In general, the adaptation of international students within the Chinese university education system falls under the category of academic adaptation within cross-cultural adaptation.

The study of cross-cultural adaptation began in the early 20th century in the United States, initiated by American anthropologists Robert Redfield, Ralph Linton, and Melville Herskovits. Redfield et al. (1936) believed that cross-cultural adaptation involves individuals and is a result of continuous interaction between two different cultures, leading to changes in cultural patterns. Early cross-cultural studies were primarily conducted from anthropological and sociological perspectives, focusing on identifying differences between cultures. Today, research and explanations of cross-cultural adaptation have expanded into other disciplines, with academic adaptation, including educational adaptation, being studied as a distinct field.

The earliest research on the concept of academic adaptation abroad was conducted by Tinto (1975). He proposed a longitudinal model of college student dropout, which posits that universities have two subsystems: an academic system and a social system. The academic system encompasses students ‘academic performance and intellectual development, while the social system includes peer-group interactions and teacher-student interactions. Baker and Siryk (1984) introduced the concept of academic adaptation earlier, defining it as the ability of international students to successfully manage their professional studies and meet the demands of the educational system. Ballard (1987) defined academic adaptation as the ability of international students to adapt to the academic life in their host country, including the teaching and learning styles of local universities. Ward et al. (2001) and others further clarified the standards for academic adaptation, emphasizing that academic achievement is the primary measure and central focus for international students pursuing a degree. Kaur (2007) defined academic adaptation as the changes students make to promote academic progress when facing different educational systems. Dunn (2006) developed an academic adjustment framework for international students, building on Pascarella and Terenzini’s research on the educational attainment of American college students. This framework outlines that the academic adjustment of international students includes academic specialization, English proficiency, academic achievement, accommodation, and extracurricular activities. Ramsay et al. (1999) and others, building on Anderson’s concept of cross-cultural adaptation, defined academic adaptation as the “alignment between learners and their academic environment.” Feng and Li (2002) define academic adaptation as the psychological and behavioral process where individuals adjust themselves to meet the needs of the environment and learning, aiming to achieve a balance between the individual and the environment. Ryan (2005) suggests that academic adaptation involves adapting to the unique academic learning requirements of the host country, which includes the teaching and learning styles of the host country. Cheng and Fox (2009) note that academic adaptation refers to the dynamic process of different language and cultural students in English-medium universities adapting to the academic learning culture.

Research on the academic adaptation of international students in China started relatively late, and in the early stages, there was no unified term for the subjects of study, whether they were domestic primary and secondary school students, college students, or international students. Recent Chinese literature mostly refers to the academic adaptation of international students as ‘academic adaptation,’ a term adopted in this article. Zhu (2011) defined the academic adaptation of international students as ‘the process of integrating international students into the academic and social systems of host country universities.’ The former refers to the academic performance and intellectual development of international students, while the latter involves their interactions with other school members, including peer relationships, teacher-student relations, and participation in club activities. Completing studies and dropping out are two outcomes of academic adaptation. Additionally, Zhu (2008) used the term ‘educational adaptation’ to study the adaptation of Chinese degree and exchange students to the foreign educational environment. This study defines academic adaptation as the continuous adjustment and mutual transformation between international students and the academic environment of host country universities until they achieve a harmonious fit.

Currently, there is no unified definition or research dimension for ‘academic adaptation’ in the academic community, and there is no universal scale for measuring the academic adaptation of international students in China. Since most international students are college or graduate students, many scholars adapt the College Student Adaptation Scale (SACQ) to measure the academic adaptation of international students. Some widely recognized scales for measuring college student academic adaptation include the College Student Adaptation Scale (SACQ), the Freshman College Student Adaptation Scale (CAS), and the Chinese College Student Learning Adaptation Scale. Scholars from China and abroad have developed different academic adaptation scales for international students based on their research objectives. Anderson (1994) and others developed a scale to measure the learning adaptation of short-term students, known as the Academic Adjustment Scale (AAS), which includes three dimensions: academic lifestyle, academic achievement, and academic motivation. The concept of academic lifestyle refers to the individual’s adaptation to the temporary role of a student; academic achievement refers to satisfaction with the academic process and accomplishments; and academic motivation refers to the driving force that motivates students to continue and complete their studies. Dunn (2006) identified 10 factors related to the academic adaptation of Chinese students in the United States: major, language, academic achievement, accommodation, extracurricular activities, peer relationships, teacher-student relationships, pre-departure preparation, financial situation, self-perception, and personal experience.

Some scholars have developed an academic adaptation scale for international students based on the one for college students. Zhu (2011) categorized the academic adaptation of international students into several aspects: curriculum design, learning content, learning methods, evaluation, teacher-student relationships, peer relationships, extracurricular activities, and rules and regulations. Specific issues include attending classes regularly, selecting courses they are interested in, understanding classroom content, completing assignments on time, taking notes in class, participating in discussions, focusing on studies, adapting to Chinese teaching methods, understanding Chinese grading systems, engaging in extracurricular activities, interacting with school administrators, and befriending students from their home country. Liu and Jia (2014) identified three main areas of academic adaptation for international students in China through interviews: curriculum planning, course content, and teaching methods. Xie (2017) examined the academic adaptation of international students from multiple perspectives, including curriculum, evaluation, learning attitude, course content, extracurricular activities, curriculum design, classroom environment, interpersonal interactions, rules and regulations, teacher-student interactions, and classroom settings. Liao et al. (2021) divided the academic adaptation of international students into several dimensions: language proficiency, teaching methods, curriculum, learning strategies, and library use.

A thorough examination of the dimensions defined by various researchers reveals that the core aspects are consistent, all involving individual and environmental factors. However, due to differences in research objectives and methods, each researcher focuses on different aspects. This study focuses on Southeast Asian international students and categorizes academic adaptation into two dimensions: academic and social, which are further divided into 11 sub-dimensions. The academic dimension includes specific aspects such as learning attitude, motivation, methods, and school teaching, while the social dimension includes aspects such as social relationships.

Scholars both domestically and internationally have also paid significant attention to the factors influencing academic adaptation. There are numerous factors that influence students’ learning adaptation, and different researchers have explored these from various perspectives, including self-esteem, academic self-efficacy, learning attitude, and physical health. Scholars from China and abroad generally agree that the main factors influencing learning adaptation include:

The impact of language proficiency. One of the most significant factors affecting international students ‘academic adaptation is their language proficiency. Research by Barratt and Huba (1994), Crano and Crano (1993), and Lewthwaite (1996) shows that language proficiency significantly influences students’ academic adaptation. Heikenheimo and Shute (1986) and others found that many Asian international students face challenges such as understanding lectures, taking notes, answering questions, and writing papers. White and Brown (1983) noted that language proficiency poses a significant challenge for international students. Whether they are engaged in any activities, the level of language proficiency determines whether international students can smoothly integrate into life and social interactions in China. Liu (2012) identified differences in teaching and learning, as well as language and communication, as the two primary reasons for academic maladjustment among Chinese-African international students. Jiang (2016) also highlighted that language proficiency is a crucial factor. Ai (2016) found that African and Asian international students studying in China encounter difficulties in their academic performance, including language barriers, interaction with professors, communication with mentors, and interactions with classmates. In these studies, academic adaptation is analyzed as a cross-cultural factor.

The allocation of lecturers and mentors. Teachers also play a significant role in the academic adaptation of international students. Research by Powell (2003), Robertson et al. (2000), and Ryan (2005) has shown that there is a mismatch between the learning expectations of international students and the teaching methods of instructors. This mismatch makes it challenging for teachers to meet the academic needs of international students. Liu and Pang (2004) explored from the students ‘perspective how teachers’ teaching methods affect their academic adaptation. Students expressed a desire for more interaction with their teachers to enhance their learning experiences.

The influence of learning attitude. Chartrand (1990) showed that students’ self-evaluation, sense of responsibility for learning and learning expectations had a great impact on their academic adaptation.

Purpose of studying abroad. Chirkov et al. (2007) pointed out that the motivation of students studying abroad is a factor that can predict whether they adapt to foreign life. His research on Chinese students in Canada and Belgium showed that the motivation of studying abroad has an important impact on the cross-cultural adaptation of students.

The impact of individual factors. People of different ages exhibit significant differences in academic adaptation. Gaither and Griffin (1971) found that younger international students encounter fewer learning adaptation issues compared to older ones. Martin’s research, which examined the relationship between students’ academic self-efficacy and optimism about academic achievement and their learning adaptability and academic performance, revealed a significant positive correlation between academic self-efficacy and students ‘academic expectations and learning adaptability levels.

The impact of major. The academic adaptation challenges faced by international students vary depending on their majors. Meloni (1984) found that students majoring in anthropology and sociology face more academic adaptation issues compared to those in engineering. Bunz (1997) found that students majoring in international relations perform better in cross-cultural adaptation than those in other majors. Nettles et al. (1986) and others discovered that factors such as the size of the school, the racial composition of the student body, the desire for a college degree, academic preparation before entering college, study habits, satisfaction with peer relationships, academic integration, and perceptions of discrimination all influence students’ academic behavior.

In addition, Chinese scholars believe that the factors affecting the academic adaptation of international students in China also include:

Differences in teaching methods (educational philosophies). Due to differences in geographical environment, social culture, and religious beliefs, different countries adopt varying teaching methods. According to Huang (2009) research, international students studying in China often find it challenging to adapt to the teaching methods of Chinese universities. Yao (2012) survey found that international students at Nanchang University struggle with the teaching styles of Chinese instructors in the classroom.

The impact of learning ability. Individual factors of learners in language teaching can affect the effectiveness of instruction. Individual differences also influence the adaptation process of international students. Li (2014) analyzed the learning adaptation of Changchun international students in China and found several issues, such as most foreign students tend to browse websites in their native language during their free time; when interacting with Chinese students, 70% of the international students prefer to guess the meaning of unclear questions rather than actively seek clarification. Zou (2014) discovered through research that students’ professional backgrounds can influence their learning adaptation.

The influence of teaching facilities. Yang (2009) used a questionnaire survey to investigate the education situation of international students in Chinese universities. The questionnaire survey involved research facilities, school libraries, campus networks, teaching materials, etc., and found that international students were not satisfied with these aspects.

The influence of curriculum setting. Zhu (2008) confirmed that the satisfaction of international students with curriculum setting would affect their academic adaptation, that is to say, a good curriculum setting could promote the learning of international students.

Teaching content, classroom atmosphere, etc. Yao (2013) found that age, educational level, and understanding of the host country’s culture can affect international students’ academic adaptation. Gao (2014) and Wang (2015) discovered that classroom atmosphere, library usage, comprehension of course content, and exam performance also play a role.

By summarizing the influencing factors from previous studies, this study categorizes the factors affecting international students ‘academic adaptation into academic and social dimensions. In subsequent survey analyses, in addition to assessing individual factors affecting international students’ academic adaptation through demographic variables, the various sub-dimensions of the scale are also used to identify the factors influencing students’ adaptation in both academic and social dimensions.

Southeast Asian students studying in China form a significant group among international students, and researching their academic adaptation is a practical necessity. However, these students face numerous challenges in adapting to both the learning environment and the Chinese education system, with various issues that need urgent attention. Moreover, most research on academic adaptation, both domestically and internationally, has been conducted primarily in developed European and American countries. While there are many studies on cross-cultural adaptation for international students, most of them focus on Western countries, particularly those from developing countries studying in developed nations. These studies often focus on issues of economic cooperation and development, with a particular emphasis on OECD member countries. Research specifically addressing the cross-cultural adaptation of international students studying in China is still underdeveloped, and studies focusing on Southeast Asian students are even rarer. The national conditions of developed Western countries differ significantly from those of China, so research findings based on these countries may not be directly applicable to the academic adaptation of international students in China, and might also be unsuitable for Southeast Asian students. Therefore, this study will focus on the academic adaptation of Southeast Asian students in China, exploring the factors and issues that influence their adaptation, and proposing practical solutions.

This study adopts two research methods, namely questionnaire survey and interview, to solve the following problems:

The overall situation of academic adaptation of undergraduate students from Southeast Asia in China and its influencing factors;
The problems encountered by undergraduate students from Southeast Asia in China during the process of academic adaptation;
Propose coping strategies for the academic adaptation problems of undergraduate students from Southeast Asia in China.

## 2 Methods

The study aims to evaluate the academic adaptation of a specific cohort of international students in China, utilizing a questionnaire survey and interviews to provide insights that could improve the support systems for these students. Presently, there is no specialized tool tailored to assess the academic adaptation of foreign students in China. Nonetheless, there are recognized and extensively used scales that measure the adaptability of college students, such as the Student Adjustment to College Questionnaire, the College Adjustment Scales, and the Adaptation Scale for Chinese College Students. Numerous researchers have adapted these scales to evaluate the academic adaptation of international students, and this questionnaire follows in that tradition. Chirkov et al. (2007) research on the impact of motivation to study abroad on the cross-cultural adaptation among Chinese students in Canada and Belgium revealed that the motivation for studying abroad is a pivotal factor in determining the adaptability of international students. When constructing the academic adaptation scale, the academic adaptation questionnaire by Zhu (2011) and the socio-cultural adaptation scale (SCAS) proposed by Ward and Kennedy (1999) were referenced.

The survey is divided into four sections: demographic information, motivation for studying abroad, academic adaptation, and life adaptation. The demographic section includes 9 items, such as gender, age, nationality, student status, duration of stay, prior experience abroad, familiarity with China, awareness of the university, and Chinese language proficiency. The motivation for studying abroad section consists of eleven items, including statements like “I must study in China” and “the favorable university living and working conditions (e.g., facilities, library, etc.).” The academic adaptation scale comprises 49 items, utilizing a Likert 5-point scale ranging from 1 (lowest) to 5 (highest), and the life adaptation scale has 23 items. The respondents were mainly Southeast Asian students in their third and fourth years at H University.

The survey was conducted online from April 27 to May 17, 2022. A total of 160 questionnaires were distributed, with 160 returned and 143 considered valid, resulting in a valid response rate of 89.4%. The data was then analyzed using SPSS21 to interpret the survey results. The Cronbach’s Alpha of 0.859 and the KMO value of 0.742 indicate that the study motivation scale has excellent internal consistency and robust structural validity. Similarly, the academic adaptation scale demonstrates a Cronbach’s Alpha of 0.959 and a KMO value of 0.892, signifying superior internal consistency and strong structural validity. Lastly, a Cronbach’s Alpha of 0.961 and a KMO value of 0.929 for another scale confirm its excellent internal consistency and commendable structural validity.

Among the 143 respondents, 68.5% identified as female and 31.5% as male. The ethnic breakdown was as follows: 40.6% were Chinese, 11.9% were international Chinese, and 47.6% were non-Chinese foreigners. A significant majority, 90.9%, of the students were aged between 20 and 23 years old. The students primarily hailed from Thailand (39.9%), Indonesia (35.7%), Laos (12.6%), the Philippines (8.4%), and Myanmar (3.5%). Regarding the duration of their stay abroad, 38.5% of the international students had been in the country for 1–2 years, 37.8% for more than 3 years, and 23.8% for 2–3 years. In terms of their proficiency in Mandarin, as measured by the HSK levels, 9.1% of international students were at levels 1–2, 66.4% were at levels 3–4, and 24.5% were at levels 5–6. Among the 8 randomly selected interviewees, there were an equal number of boys and girls, with 5 juniors and 3 seniors. The interviewees represented a mix of nationalities: 3 from Indonesia, 2 from Thailand, 2 from Myanmar, and 1 from the Philippines.

## 3 Findings

Based on Table 1, the overall average value for academic adaptation is 3.50, with a score of 3, indicating that the average level of academic adaptation among undergraduate students at H University and in Southeast Asia studying in China is moderate. Notably, the maximum value reaches 4.78, while the minimum value is 2.67. This suggests that while some students exhibit a relatively high level of academic adaptation, others demonstrate a lower level, resulting in a considerable overall variance in academic adaptation among the students.

**Table 1:** Mean values of academic adaptation.

Mean values	N	Min	Max	Mean
Comprehensive academic adaptation	143	2.67	4.78	3.50
N	143			

### 3.1 Level of academic adaptation across all dimensions

The study presents the levels and rankings of academic adaptation for each dimension, aiming to understand the distinct academic adaptation levels of each dimension and the overall academic adaptation across the ten dimensions. The hierarchy of the average academic adaptation levels within each dimension is depicted in Table 2 below.

**Table 2:** Ranking of mean values of academic adaptation levels across ten dimensions.

Item	Dimension	Mean	Item	Dimension	Mean
1	Psychological status	3.10	6	social relationship	3.59
2	Learning motivation	3.34	7	school teaching	3.60
3	Learning attitude	3.42	8	learning ability	3.60
4	Learning methods	3.49	9	school management	3.63
5	Academic environment	3.56	10	Chinese level	3.66

The data showcased in Tables 1, 2 depict the comprehensive academic adaptation levels of Southeast Asian students and the average scores for each dimension of academic adaptation. In general, the average academic adaptation score for international students in China is 3.50, signifying a moderate level of adaptation. The average scores for the ten dimensions of academic adaptation, arranged from highest to lowest, are as follows: Chinese language proficiency, school management, instructional quality, learning ability, social interactions, learning environment, study methods, learning attitude, learning motivation, and psychological well-being. Notably, the average scores for academic adaptation in the four dimensions of psychological status, learning motivation, learning attitude, and study methods fell below the overall average.

The survey indicated that Southeast Asian international students encountered the most significant challenges in academic adaptation within the psychological status dimension, with an average score of 3.10, the lowest across all dimensions. This implies that psychological issues constitute the most substantial obstacle to their academic adjustment. Upon closer inspection of the psychological status subcategories, it was observed that the mean score for item 2, “psychological anxiety,” was notably low at 2.77, whereas the mean score for item 3, “I feel lonely in China,” was relatively elevated at 3.39. Considering that items 2 and 3 are inversely worded, it appears that Southeast Asian students are experiencing significant psychological distress and feelings of loneliness during their studies.

In terms of academic adaptation within the learning motivation dimension, international students achieved an average score of 3.34, which falls below the overall average for academic adaptation. This dimension is among the ten scoring lowest, suggesting there is potential for enhancement in their motivation to learn. A detailed examination of the specific items reveals that item 12, “I often actively read professional books,” had the lowest average score at 3.10, while item 11, “I have a clear career choice for the future, study hard,” had the highest average score at 3.59.

The average academic adaptation of international students, particularly in terms of learning attitude, is 3.42, which is slightly below the overall academic adaptation level. A detailed analysis of the average values for learning attitudes indicates that sub item 7, “I believe one must pass each subject,” has the lowest mean value at 3.00. In contrast, sub item 8, “I find college study very interesting,” has the highest mean value at 3.84.

In the dimension of learning method, the average academic adaptation of international students is 3.49, which is also slightly below the overall academic adaptation level. A specific analysis reveals that the lowest average (3.38) pertains to “I will preview (or review after class).” Item 18, “When I encounter difficulties, I will seek assistance from my teachers or classmates,” has the highest average value at 3.70.

The average level of academic adaptation within the learning ability dimension is 3.60, surpassing the overall academic adaptation level and indicating a commendable level of proficiency. This implies that international students demonstrate a robust adaptation in terms of their learning capabilities. Upon closer inspection of specific items, item 33, “My strong independent learning ability,” exhibits the lowest mean value at 3.36, whereas item 30, “I can pass the exam,” boasts the highest mean value at 3.92. The project’s mean span is notably wide.

In the context of international students’ individual learning dimensions, it has been observed that the mean score for learning ability, at 3.60, surpasses the overall average for academic adaptation, which stands at 3.50. Conversely, dimensions such as learning attitude, learning motivation, and learning methods are found to be below the average level of overall academic adaptation. Nevertheless, each of these dimensions has an average score greater than 3, indicating that students are performing at an average level in these areas. This suggests that there is potential for improvement in how these individual learning dimensions are related to academic adaptation.

The average academic adaptation of international students to the school environment is 3.56, slightly exceeding the overall academic adaptation level. This suggests that international students are generally well integrated into the learning environment. Upon closer inspection of specific sub-items, it becomes apparent that item 36, “I can adapt to the management of Chinese universities,” has the lowest average score of 3.59, whereas item 37, “I can adapt to the schedule of Chinese universities,” boasts the highest average score at 3.65.

The average level of academic adaptation among international students in the social relationship dimension is 3.59, exceeding the overall academic adaptation benchmark. This suggests that their adaptation to social relationships within the academic context is commendably high. Specifically, sub-item 45, “No obstacles in interacting with school office staff,” has the lowest average score at 3.45, whereas sub-item 43, “I maintain a good rapport with classmates and am well-integrated into the class,” boasts the highest average score of 3.69.

In the university teaching dimension, international students demonstrate an average academic adaptation level of 3.60, surpassing the overall academic adaptation benchmark. This indicates that they are successfully adapting to the university’s teaching methodologies. Sub-item 25, “I can adjust to the teachers’ grading system,” has the lowest mean score at 3.50, whereas sub-item 22, “I am content with the teachers’ instructional approach,” boasts the highest mean score of 3.76.

Furthermore, the average adaptation of international students to the school management dimension is 3.63, which surpasses the overall academic adaptation level, suggesting a generally positive adjustment to the management aspects of the university. Sub-item 36, “I can adjust to the management style of Chinese universities,” has the lowest average score at 3.59, whereas sub-item 37, “I can adjust to the working hours of Chinese universities,” boasts the highest average score at 3.65.

The average score for international students’ academic adaptation, particularly in terms of their proficiency in the Chinese language, stands at 3.66, exceeding the overall academic adaptation level. When contrasting with other dimensions, it is evident that their adaptation is most pronounced in the realm of Chinese language abilities. The sub-item with the lowest average score is item 47, “Can understand Chinese textbooks,” with an average of 3.57. In contrast, item 48, “Able to complete homework assigned in Chinese by the teacher,” boasts the highest average score at 3.76.

Overall, international students in China exhibit a positive adaptation to the school environment and teaching management, indicating that the academic challenges they encounter mainly originate from the learning process itself.

### 3.2 Differences in academic adaptation

Based on the questionnaire survey of the respondents from the personal survey results, the study investigated the academic adaptation of Southeast Asian students in China by examining various factors such as the respondents’ gender, age, nationality, student status, identity, duration of learning Chinese, experience of studying abroad, understanding of China, perception of the university, and Chinese language proficiency. One-way ANOVA was also employed to assess the differences in academic adaptation among Southeast Asian undergraduate students in China.

Table 3 presents the ANOVA results for gender factors, with a *p*-value of 0.554, which exceeds 0.05. This suggests that the differences in academic adaptation among Southeast Asian students are not statistically significant.

**Table 3:** Gender: one-way ANOVA.

Gender	SS	df	M.S.	F	Sig
Between-group	188.433	1	188.433	0.351	0.554
Within-group	75685.105	141	536.774		
SST	75873.538	142			

Table 4 indicates that, among the age-related factors, the *p*-value was 0.712, which exceeds 0.05. This suggests that there is no statistically significant difference in academic adaptation among international students in China based on their age.

**Table 4:** Age: one-way ANOVA.

Age	SS	df	M.S.	F	Sig
Between-group	3410.189	9	378.910	0.695	0.712
Within-group	72463.350	133	544.837		
SST	75873.538	142			

The respondents’ nationalities span five countries: Thailand, Laos, Myanmar, Indonesia, and the Philippines. Table 5 indicates a *p*-value of 0.017, which is less than 0.05, suggesting that the variance in nationality among Southeast Asian students is statistically significant. According to Table 6, the data reveal that Myanmar leads with the highest academic performance among Southeast Asian students, followed by Indonesia, the Philippines, Thailand, and Laos.

**Table 5:** Nationality: one-way ANOVA.

Nationality	SS	df	M.S.	F	Sig
Between-group	6272.999	4	1568.250	3.109	0.017
Within-group	69600.539	138	504.352		
SST	75873.538	142			

**Table 6:** Nationality: mean value and standard deviation.

Nationality	N	Mean	S.D.
The Philippines	12	176.750	26.9347
Thailand	57	166.439	21.3675
Indonesia	51	177.098	22.3967
Laos	18	162.944	20.2178
Burma	5	189.200	31.7128
Total	143	171.462	23.1154

Table 7 presents the results of the ANOVA analysis for student identity, with the data revealing a *p*-value of 0.004, which is less than the 0.05 threshold. This suggests a statistically significant difference in the academic identity among Southeast Asian students in China. Table 8 outlines the mean values and standard deviations for the academic adaptation levels of students across various identities. The findings indicate that Chinese students exhibit a markedly superior level of academic adaptation compared to both international Chinese students and non-Chinese international students.

**Table 7:** Student identity: one-way ANOVA.

Student identity	SS	df	M.S.	F	Sig
Between-group	5766.252	2	2883.126	5.757	0.004
Within-group	70107.286	140	500.766		
SST	75873.538	142			

**Table 8:** Student identity: mean and standard deviation.

Student identity	N	Mean	S.D.
Foreign citizens of Chinese origin	58	178.448	22.8620
Overseas Chinese nationals	17	159.824	19.8502
Foreign citizens of non-Chinese descent	68	168.412	22.5304
Total	143	171.462	23.1154

The respondents had studied Chinese for durations ranging from 1–2 years, 2–3 years, to more than 3 years. Table 9 presents the results of the ANOVA analysis on the length of time spent learning Chinese. The *p*-value obtained was 0.753, which exceeds the conventional significance level of 0.05, suggesting that the duration of Chinese language study does not significantly impact the academic adaptation of international students.

**Table 9:** Duration of Chinese language learning: one-way ANOVA.

Duration of Chinese language learning	SS	df	M.S.	F	Sig
Between- group	306.323	2	153.162	0.284	0.753
Within- group	75567.215	140	539.766		
SST	75873.538	142			

Table 10 presents the variance analysis of international experience, where the p-value of 0.322 exceeds 0.05, indicating that there is no significant impact on the academic adaptation of international students in China.

**Table 10:** Going abroad experience: one-way ANOVA.

Going abroad experience	SS	df	MS	F	Sig
Between-group	528.367	1	528.367	0.989	0.322
Within-group	75345.172	141	534.363		
SST	75873.538	142			

Table 11 presents the variance analysis of international students’ knowledge of China, with a p-value of 0.463 exceeding the threshold of 0.05, indicating that there is no statistically significant correlation between their understanding of China and their academic adaptation in the country.

**Table 11:** Understanding about China: one-way ANOVA.

Understanding about China	SS	df	MS	F	Sig
Between-group	829.316	2	414.658	0.774	0.463
Within-group	75044.222	140	536.030		
SST	75873.538	142			

Table 12 presents the variance analysis for the extent of international students’ academic adaptation, revealing a *p* value of 0.075, which exceeds the threshold of 0.05. This indicates that there is no statistically significant correlation between the degree of Southeast Asian students’ adaptation and their academic performance.

**Table 12:** Understanding about universities: one-way ANOVA.

Understanding about universities	SS	df	M.S.	F	Sig
Between-group	2751.121	2	1375.560	2.634	0.075
Within-group	73122.418	140	522.303		
SST	75873.538	142			

The survey classified the Chinese language proficiency of international students into three levels: HSK 1–2, HSK 3–4, and HSK 5–6. Table 13 displays the analysis of variance for Chinese language proficiency, where the p-value of 0.00, being less than 0.05, indicates a statistically significant difference in academic adaptation and Chinese language proficiency among international students. Examining the mean values and standard deviations in Table 14, it is clear that as proficiency in the Chinese language increases, so does the level of academic adaptation among international students.

**Table 13:** Chinese level: one-way ANOVA.

Chinese level	SS	df	M.S.	F	Sig.
Between-group	9991.714	2	4995.857	10.616	0.000
Within- group	65881.825	140	470.584		
SST	75873.538	142			

**Table 14:** Chinese level: mean value and standard deviation.

Chinese level	N	Mean	S.D.
HSK1-2	13	159.692	15.9655
HSK 3–4	95	167.874	22.1216
HSK4-5	35	185.571	22.2437
Total	143	171.462	23.1154

A thorough examination of the aforementioned factors indicates that students’ nationality, student status, and proficiency in Chinese have a significant impact on their academic adaptation. On the other hand, there is no significant variance in academic adaptation among students based on gender, age, length of Chinese language study, studying abroad, comprehension of Chinese culture, or understanding of the university. For more detailed information, please refer to Table 15.

**Table 15:** Academic adaptation differences in congenital individual factors.

Congenital individual factors	Significant difference	Congenital individual factors	Significant difference
Gender	×	going abroad experience	×
Age	×	understanding about China	×
Nationality	√	understanding about universities	×
Student identity	√	Chinese level	√
Duration of Chinese language learning	×		

“×” represents no significant difference; “√” represents a significant difference.

## 4 Analysis

### 4.1 Impact of individual influencing factors on academic adaptation

Individual influencing factors encompass both innate and acquired elements. Innate factors primarily pertain to the nine demographic variables. As indicated in Table 15, nationality, student status, and proficiency in the Chinese language significantly influence the academic adaptation of international students.

The acquired factors encompass dimensions pertinent to academic adaptation and personal aspects, including psychological status, learning attitude, methodology, motivation, ability, and proficiency in Chinese. The correlations between these acquired individual factors and academic adaptation are detailed in Table 16.

**Table 16:** Correlations of acquired individual influencing factors with academic adaptation.

Individual influencing factors		Academic adaptation
Psychological status	Pearson correlation	0.237**
	Significance (Bilateral)	0.004
Attitude to learning	Pearson correlation	0.726**
	Significance (Bilateral)	0.000
Learning motivation	Pearson correlation	0.745**
	Significance (Bilateral)	0.000
Learning methods	Pearson correlation	0.744**
	Significance (Bilateral)	0.000
Learning ability	Pearson correlation	0.892**
	Significance (Bilateral)	0.000
Chinese level	Pearson correlation	0.801**
	Significance (Bilateral)	0.000

** Indicates that the correlation is significant at the 0.01 level (*p* < 0.01).

The study uncovers a significant and positive correlation between the psychological well-being of international students and their level of academic adaptation. Put differently, improvements in the students’ psychological status are accompanied by enhanced academic adaptability. The correlation coefficient for these two variables stands at 0.237, situated within the range of 0.2 to 0.4, indicating a moderate association between the psychological well-being and academic adaptation of international students. It is worth noting that this correlation is the weakest among several other individual factors that were considered.

Upon examining the dimension of academic adaptation within the psychological status, it was observed that students exhibit the lowest mean value in this area, primarily due to psychological anxiety stemming from learning challenges. Through interviews, I discovered that international students experience considerable stress during course studies and thesis writing. Interviewer 7 mentioned, “During my sophomore year, I felt immense pressure because the coursework was slightly more challenging than in my freshman year. I struggled to adapt, which led to significant stress and uncertainty about how to cope.” Interviewer 8 expressed that the process of writing his graduation thesis was particularly painful, as he worried about his ability to graduate and often suffered from sleepless nights.

As illustrated in Table 16, the correlation coefficients between academic adaptation and various factors such as learning attitude, motivation, methods, ability, and proficiency in Chinese all exceed 0.7, indicating a robust positive correlation. This suggests that the more positive a student’s learning attitude, the stronger their motivation, the more effective their learning methods, the greater their learning ability, and the higher their proficiency in Chinese, the greater their level of academic adaptation will be.

### 4.2 Impact of environmental influencing factors on academic adaptation

The environmental factors that impact academic adaptation encompass various dimensions related to the external environment, as outlined in the academic adaptation scale. These dimensions consist of school teaching, school management, the learning environment, and social relationships. The interplay among these diverse environmental factors and their influence on academic adaptation are elaborated in Table 17 below.

**Table 17:** Correlations between environmental influencing factors and academic adaptation.

Environmental influencing factors		Academic adaptation
School teaching	Pearson correlation	0.877**
	Significance (Bilateral)	0.000
School management	Pearson correlation	0.818**
	Significance (Bilateral)	0.000
Academic environment	Pearson correlation	0.837**
	Significance (Bilateral)	0.000
Social relationship	Pearson correlation	0.790**
	Significance (Bilateral)	0.000
	Significance (Bilateral)	0.000

** Indicates that the correlation is significant at the 0.01 level (*p* < 0.01).

As evidenced by the data in Table 17, there exists a significant positive correlation among school teaching, school management, learning environment, social relationships, and the level of academic adaptation among students. In other words, enhancements in the quality of teaching, management, and the learning environment, coupled with more robust social relationships, are linked to a higher level of academic adaptation in international students.

### 4.3 Influence of motivation to study abroad on academic adaptation

To comprehend the comprehensive motivation driving international students to seek education overseas, the intensity coefficient was utilized to evaluate the respondents’ motivational strength. The intensity coefficient signifies the ratio of the total score to the maximum possible score for average study abroad motivation. A coefficient under 0.20 indicates a weak investigation of study motivation, ranging from 0.2 to 0.4 suggests a moderate level of motivation, while a coefficient from 0.4 to 0.6 signifies a strong motivation. Values between 0.60 and 0.80 denote a very strong motivation, and those between 0.80 and 1.00 indicate an extremely strong motivation. Data analysis indicates that the average motivational intensity for undergraduate students from Southeast Asia is 0.75, pointing to a strong motivation. Among the respondents, 1.4% displayed weak motivation, 18.9% moderate, 46.9% strong, and 32.8% very strong.

#### 4.3.1 Analysis of the motivation factors of study abroad

To explore the diverse motivations that propel international students to continue their education in China, researchers conducted a factor analysis on the initial 11 variables related to these motivations, utilizing SPSS version 21.0. After several iterations and the removal of the superfluous variables “scholarship” and “Xiamen attracted me,” it was determined that two factors surfaced with eigenvalues surpassing 1, explaining a total variance of 61.261%.(Table 18).

**Table 18:** Analysis of the influencing factors of motivation for studying abroad.

Component	Initial eigenvalue			Extraction sums of squared loadings			Rotation sums of squared loadings		
	Total	% of variance	Cumulative%	Total	% of variance	Cumulative%	Total	% of variance	Cumulative%
1	5.662	51.472	51.472	5.662	51.472	51.472	4.682	42.566	42.566
2	1.077	9.790	61.261	1.077	9.790	61.261	2.056	18.695	61.261
3	0.944	8.584	69.845						
4	0.707	6.426	76.271						
5	0.568	5.161	81.432						
6	0.501	4.551	85.982						
7	0.447	4.060	90.042						
8	0.272	2.473	96.260						
9	0.172	1.568	100.000						

As depicted in Table 19, after several iterations and the removal of extraneous items, such as item 1 “scholarship” and item 11 “Xiamen attracts me,” the first factor unites the five initial variables—items 3, 4, 5, 7, and 8—with a factor loading of at least 0.695. These variables encompass the desire to “enhance my Chinese language abilities and gain insight into Chinese culture,” “study specialized subjects in Chinese, such as Chinese painting,” “the educational experience at H university will aid me in future employment,” “obtain exceptional opportunities to travel within China and deepen my cultural understanding,” and “exercise my capacity to navigate unfamiliar cultural contexts.” This factor can be designated as “internal motivation for studying abroad,” capturing the inherent motivations that drive international students to seek education internationally. The second factor, with a factor loading of at least 0.524, consolidates four original variables from projects 2, 6, 9, and 10, which are “the living and working conditions at H university (including facilities, library, etc.) surpass those at Chinese universities,” “parents/friends have recommended that I study at H university,” “it is more convenient to apply to key Chinese universities from H university,” and “my friends at H university are studying.” This factor can be labeled “external motivation for studying abroad,” signifying the reasons international students opt to study in China due to external factors.

**Table 19:** Rotated component matrix for studying abroad motivations.

Motivations for studying abroad	Component	
	1	2
2. Good living and working conditions	0.104	0.743
3. Improve my Chinese language proficiency	0.878	0.116
4. Learn from Chinese expertise	0.695	0.246
5. Learning experience is good for employment	0.812	0.306
6 Parents and friends suggest it	0.338	0.657
7 Travel in China to understand Chinese culture	0.747	0.342
8 Cultivate individual abilities	0.828	0.170
9 The application process is easy	0.178	0.524
10 Friends are at H University	0.025	0.888

#### 4.3.2 Main motivation factors affecting academic adaptation

As illustrated in Tables 20, a significant positive correlation exists between the factors of studying abroad motivation and academic adaptation, suggesting that the motivational opportunities associated with studying abroad have an impact on the academic adaptation levels of students from Southeast Asia. The correlation coefficients reveal that, in comparison to factor 2, “external motivation,” factor 1, “internal motivation,” exhibits a stronger correlation with academic adaptation. This indicates that enhancing the internal motivation of international students can facilitate further enhancements in their academic adaptation levels.

**Table 20:** Correlation between diverse factors of motivation for studying abroad and academic adaptation.

Motivation for studying abroad		Academic adaptation
Factor 1 internal motivation to study abroad	Pearson correlation	0.571**
	Significance (Bilateral)	0.000
	N	143
Factor 2 external motivation to study abroad	Pearson correlation	0.197*
	Significance (Bilateral)	0.019
	N	143

** Indicates that the correlation is significant at the 0.01 level (*p* < 0.01). * Indicates that the correlation is significant at the 0.05 level (*p* < 0.05).

#### 4.3.3 Impact of life adaptation on academic adaptation

As illustrated in Table 21, the overall average score of Southeast Asian students in China is 2.59, which falls below the midpoint of 3, suggesting that the performance of international students in China is on the lower end.

**Table 21:** Overall mean of life adaptation.

Life adaptation	N	Min	Max	Mean
Overall life adaptation	143	1.00	5.00	2.59
N	143			

To explore the multifaceted aspects of life adaptation among international students in China, researchers utilized SPSS 21.0 to examine the 11 initial variables linked to learning motivation. Table 22 indicates the analysis uncovered three primary factors, each with eigenvalues surpassing 1, accounting for a total variance of 67.992%.

**Table 22:** Analysis of life adaptation influencing factors.

Component	Initial eigenvalue			Extraction sums of squared loadings			Rotation sums of squared loadings		
	Total	% of variance	Cumulative %	Total	% of variance	Cumulative %	Total	% of variance	Cumulative %
1	12.56	54.608	54.608	12.56	54.608	54.608	6.332	27.529	27.529
2	1.859	8.081	62.689	1.859	8.081	62.689	4.854	21.103	48.632
3	1.22	5.303	67.992	1.22	5.303	67.992	4.453	19.359	67.992
4	0.962	4.181	72.172						
5	0.798	3.47	75.642						
6	0.766	3.329	78.972						
7	0.641	2.786	81.758						
8	0.517	2.247	84.005						
9	0.457	1.986	85.992						
10	0.422	1.833	87.825						
11	0.376	1.633	89.457						
12	0.333	1.449	90.906						
13	0.323	1.402	92.309						
14	0.287	1.246	93.555						
15	0.257	1.118	94.672						
16	0.22	0.957	95.63						
17	0.195	0.849	96.478						
18	0.188	0.816	97.295						
19	0.175	0.76	98.055						
20	0.139	0.603	98.658						
21	0.12	0.522	99.181						
22	0.112	0.485	99.666						
23	0.077	0.334	100						

Table 23 indicates that the first factor encompasses nine original variables.: item 02, 03, 04, 05, 06, 07, 08, and 09, each with a loading of at least 0.405. These encompass various aspects, including “adapting to China’s diet,” “adapting to China’s climate,” “adapting to China’s transportation,” and other elements. This factor is designated as “adaptation to the external natural and social Environment.”

**Table 23:** Rotated component matrix for life adaptation influencing factors.

Life adaptation influencing factors	Component		
	1	2	3
01. Diet	0.787	0.284	0.298
02. Climate	0.786	0.237	0.333
03 Transportation	0.755	0.195	0.328
04. Shopping environment	0.722	0.299	0.248
05. The pace of life	0.709	0.257	0.401
06. Local accent	0.700	0.324	0.325
07. Use of the Chinese language	0.679	0.390	0.150
08 The Chinese culture and the Chinese people	0.657	0.312	0.257
09. Local price levels	0.529	0.367	0.253
10. Make local friends	0.131	0.810	0.167
11. Activities held by the Chinese people, such as parties, etc.	0.340	0.766	0.144
12. The Chinese people are genuine and polite	0.373	0.764	0.201
13. Chinese humor	0.237	0.664	0.211
14. Chinese restaurant and store services	0.231	0.742	0.227
15. Service provided by Chinese hospitals and banks and post offices	0.362	0.667	0.168
16. Public services delivered by China’s administrative agencies	0.306	0.653	0.235
17. Social and public order in China	0.388	0.242	0.738
18. Chinese hygiene habits	0.354	0.145	0.734
19. Religious and culture	0.157	0.251	0.670
20. Laws and regulations	0.396	0.178	0.767
21. The dormitory management rules	0.405	0.133	0.662
22. Get along with roommates	0.349	0.177	0.608
23. Get along with international students from other countries	0.306	0.339	0.538

The second factor encompasses seven original variables: item10, 11, 12, 13,14, 15, and 16, each with a loading factor of no less than 0.653. It encompasses considerations such as “adapting to services at China’s restaurants and shops,” “adapting to services at Chinese hospitals, banks, and post offices,” and “adapting to the administrative structure of China, such as college management offices.” This factor is named “adaptation to the external service environment.”

The third factor encompasses seven original variables: item 17,18,19,20,21, 22, and 23, each with a loading of at least 0.538. These encompass “compliance with Chinese laws and regulations,” “adapting to the management rules of the school’s student dormitory,” and various others. This factor is designated as “adaptation to the Chinese System, etc.”

As indicated in Table 24, the three dimensions of life adaptation exhibit significant positive correlations with academic adaptation. It is suggested that life adaptation influences the academic adaptation levels of undergraduate students from Southeast Asia studying in China. To enhance the academic adaptation of these students, it is imperative to concentrate on the challenges and difficulties they face in adjusting to life abroad.

**Table 24:** Correlation between diverse factors of life adaptation and academic adaptation.

Life adaptation influencing factors		Academic adaptation
Factor 1 adapt to the natural and social environment	Pearson correlation	0.652**
	Significance (Bilateral)	0.000
	N	143
Factor 2 adapt to the external service environment	Pearson correlation	0.541**
	Significance (Bilateral)	0.000
	N	143
Factor 3 adapt to the Chinese system, etc	Pearson correlation	0.531**
	Significance (Bilateral)	0.000
	N	143

** indicates that the correlation is significant at the 0.01 level (*p* < 0.01).

Upon analyzing the ten items with the lowest mean scores in life adaptation (refer to Table 25 below for data), five of these items (01, 02, 03, 04, 05) originate from factor 1, “adaptation to the natural and social environment.” Upon their arrival in the host country, students are confronted with cultural shock in the new setting. Their inability to adapt to China’s natural and social environment makes the issues represented by these five items particularly widespread. During interviews, most students reported experiencing initial difficulties, with some even crying daily due to homesickness. However, they indicated that over time, they gradually adapted. Interviewee 5 shared: “Initially, I struggled a lot with the food and living habits here, to the point of crying every day because I missed home so much. But after a while, I made some new friends and the teachers here have been very supportive. I’ve gradually grown accustomed to the environment and no longer feel the need to cry.”

**Table 25:** Analysis of life-adaptation means.

Item	Mean	Content
17	2.33	Social and public order in China
03	2.31	Transportation
02	2.30	Climate
14	2.27	Chinese restaurant and store services
01	2.25	Diet
05	2.24	The pace of life
21	2.22	The dormitory management rules
04	2.15	Shopping environment
22	2.14	Get along with roommates
23	2.13	Get along with international students from other countries

During the interview, interviewee 5 noted: “The university’s dormitory management is quite stringent, with weekly health inspections. Multiple failures could lead to counseling sessions and may even impact our scholarships. Additionally, we are expected to inform the counselor if we return late on several occasions.” Items 22, “Ability to harmonize with roommates,” and 23, “Ability to harmonize with international students from other countries,” scored the lowest on the life adaptation questionnaire. The impact of culture and language on the social interactions of international students appears to be relatively minor. Many international students report difficulties in getting along with their roommates. Classmate 01 shared, “Initially, I did not get along with my roommates. Even though we were from the same country, our personalities clashed. We decided to change dormitories, and the situation improved significantly.” Interviewer 4 stated, “Before I started getting along with my roommate, we had very limited communication. They are African students, and we faced a language barrier, which meant we rarely spoke. I can appreciate their religion and culture, but our living habits are vastly different, making it challenging to live together.”

Life adaptation is a key factor influencing academic adaptation, with a positive correlation between the two. Higher levels of life adaptation are associated with higher levels of academic adaptation. Among the factors, “adaptation to the social and natural environment” (Factor 1) has the highest correlation with academic adaptation, followed by “adaptation to the external service environment” (Factor 2) and “adaptation to the Chinese system” (Factor 3).

## 5 Discussion

In the post-pandemic period, international students confront multifaceted challenges in cross-cultural adaptation that adversely affect not only their academic performance but also profoundly impact psychological well-being and social integration. Higher education institutions globally have undergone rapid transitions from online to in-person instruction, generating significant adaptation stress among international student populations. For instance, mandatory campus return policies implemented by UK universities have precipitated psychological distress, financial burdens, and adverse effects on educational outcomes. Concurrently, the reduction of digital learning resources has created significant difficulties for students acclimatized to remote instruction (Zhao and Xue, 2023). The pandemic and its persistent repercussions have substantially exacerbated mental health concerns within this demographic. Empirical evidence indicates heightened levels of loneliness and academic anxiety among international students during the pandemic, further compromising cross-cultural adaptability. Particularly within the UK context, loneliness has demonstrated significant negative correlations with academic achievement and social connectivity (Dost, 2025). Furthermore, longitudinal Australian research reveals that pandemic-induced mental health deterioration among international students manifested greater severity and exhibited slower recovery trajectories compared to domestic peers (Dingle et al., 2024).

Contemporary international students encounter compounded social integration barriers. Japanese research illustrates that despite maintaining specific health protocols (e.g., hand hygiene and mask usage), international students continue to experience social isolation in quotidian interactions. Cultural disparities and linguistic barriers further intensify adaptation challenges, particularly regarding healthcare accessibility and employment support systems (Ma et al., 2024).

The pandemic has fundamentally disrupted academic trajectories and career planning. Chinese research indicates that international students increasingly prioritize innovative learning environments and cultural adaptation capacity when selecting institutions, though structural inequalities and underdeveloped innovation ecosystems remain substantial impediments. Notably, a persistent preference for distance learning modalities reflects enduring concerns regarding reintegration into traditional classroom settings (Zuo, 2022).

Mental health constitutes a fundamental pillar underpinning international students’ academic progression and personal development within the Chinese educational context. Detrimental psychological states can significantly disrupt normative learning processes and pose substantial risks to student well-being. Empirical studies indicate that international students in China generally exhibit lower levels of psychological adjustment relative to the domestic population, with mental health exerting a direct influence on academic adaptation efficacy. To facilitate international students’ adaptation within post-pandemic cross-cultural environments, higher education institutions must prioritize addressing their psychological needs during academic engagement.

Scholarly consensus advocates for the provision of specialized mental health services to mitigate experiences of loneliness and academic anxiety (Dingle et al., 2024; Dost, 2025). Consequently, institutions are advised to implement professional counseling programs designed to alleviate psychological challenges inherent in academic adaptation. Optimizing extant counseling services necessitates enhancing both the professional competence and accessibility of psychological support systems. Effective counseling provision rests upon three fundamental components: professional resource allocation, capacity for tailored service delivery, and robust institutional safeguards. Deficiencies in any of these components demonstrably compromise support efficacy.

In order to mitigate the academic adaptation challenges arising from cultural differences and value conflicts among international students in Chinese universities, it is imperative to establish a systematic framework integrating three core components: teachers’ cross-cultural sensitivity as the foundational competency; curriculum internationalization as the pedagogical vehicle; and campus inclusiveness as the environmental scaffold.

Currently, university psychological counselors encounter significant challenges pertaining to both professional competence and cultural adaptability. Many institutions recruit counselors possessing limited qualifications, predominantly focusing on general counseling while lacking specialized training for issues prevalent among international students, such as “second-language acquisition anxiety” or “cross-cultural family conflicts.” This deficit in specialized expertise impedes the ability to effectively address complex problems, including academic anxiety compounded by linguistic barriers and cultural isolation. Standardized psychological assessment instruments (e.g., SCL-90), developed within Western contexts, frequently lack localized adaptations suitable for Southeast Asian students, particularly concerning religious beliefs and collectivist cultural orientations. Such inadequacies may yield inaccurate evaluations, compromising the precision of subsequent intervention plans.

Furthermore, certain counselors manifest deficiencies in cross-cultural competence, exhibiting an inadequate comprehension of Southeast Asian cultural frameworks. Their inadequate grasp of religious taboos prevalent among Malaysian and Indonesian students, combined with a superficial understanding of collectivist values in Thailand and Laos, risks the clinical misdiagnosis of normative cultural differences as psychological disorders. For instance, student reluctance to seek assistance—potentially influenced by “face culture”—may be misinterpreted as social withdrawal. This can result in counseling programs that fail to adequately address actual needs and are deficient in requisite cross-cultural intervention capabilities. Additionally, communication barriers between Southeast Asian international students and counselors, exacerbated by students’ limited proficiency in the Chinese language, frequently hinder the comprehensive expression of psychological distress, thus obstructing the timely provision of necessary support.

The majority of universities predominantly depend on government funding for psychological counseling services. Due to the limited administrative resources, the recruitment of professional counselors is inadequate, and the ratio between counselors and international students is imbalanced. Consequently, the consultation hours and frequency are restricted, failing to meet the frequent psychological needs of international students who are confronted with cultural adaptation challenges and academic pressures.

Universities frequently assign part-time counselors in charge of international student affairs to offer counseling support. Nevertheless, these part—time staff encounter difficulties in balancing their responsibilities in student management, visa processing, and accommodation arrangements. As a result, it is arduous for them to allocate sufficient time to designing activity plans or monitoring student development.

The operational administrative processes of universities create systemic obstacles in the crisis referral efficiency of counseling services. When international students present severe psychological problems (such as depressive tendencies), they need to be referred to external mental health institutions. However, gaps in administrative procedures, including “medical insurance coordination” and “language assistance,” may postpone critical intervention opportunities.

Resource limitations, a dearth of faculty expertise, and deficiencies in administrative processes may undermine the effectiveness of support measures for international students across multiple dimensions. Specifically, these challenges can be classified into three aspects: “supply-side” constraints, “capacity limitations,” and “institutional barriers.”

On the supply side, problems such as a scarcity of counseling resources and inadequate assessment tools frequently fail to satisfy the diverse requirements of international students. In terms of capacity limitations, certain educators may lack cross-cultural competence, rendering it difficult to effectively tackle unique challenges such as language adaptation and cultural adjustment. From an institutional standpoint, inefficient administrative procedures and a lack of inter-departmental collaboration further impede the seamless implementation of support initiatives.

Consequently, rectifying existing deficiencies in psychological counseling, such as unprofessional practices and accessibility problems, constitutes a crucial step for universities to enhance the mental health and well-being of international students.

To promote the professionalization of psychological counseling, universities ought to offer specialized training programs for faculty members who have direct interactions with international students. Considering that China’s mental health services are still in the process of development and improvement, educational institutions should not solely rely on campus counselors but also engage student advisors, academic mentors, and even dormitory supervisors to provide psychological support. In addition to providing compensation or incentives for these supplementary counseling duties, institutions should place emphasis on professional development by means of language proficiency training and the enhancement of cross-cultural communication skills. Universities with a large number of Southeast Asian students could contemplate recruiting counselors with Southeast Asian cultural backgrounds and applying narrative therapy to assist students in expressing their anxiety. For instance, the use of “family story sharing” can be employed to mitigate “expectation pressure.”

Teachers act as the primary “intermediaries” through which international students interact with Chinese culture and educational systems. Their cross-cultural sensitivity directly influences the effectiveness of conflict resolution. To address this issue, schools can regularly organize cross-cultural training programs. These programs encompass cultural customs, value systems, and communication modes of various countries, which can enhance educators’ understanding of multiculturalism. For example, professional cross-cultural researchers can be invited to give lectures on cross-cultural educational philosophies and strategies to prevent conflicts resulting from misunderstandings in teaching.

In addition, arranging cultural immersion activities such as visiting international exhibitions and participating in global cultural festivals enables teachers to directly experience the allure of multiculturalism, thus enhancing their sensitivity to cultural differences. Moreover, conducting cross-cultural pedagogy seminars and maintaining reflective journals are effective means to improve teachers’ cross-cultural competence. At these seminars, educators can share cross-cultural cases encountered in their teaching practices, jointly analyze the causes, and explore adjustments to teaching methods that respect students’ cultural traditions while achieving the desired educational outcomes. Through these discussions, teachers can exchange perspectives and continuously refine their cross-cultural teaching skills.

Furthermore, establishing close mentor—mentee relationships with Southeast Asian students is of great significance. Teachers should make use of extracurricular time to communicate with students, understand their academic and personal difficulties, and offer care and support. Through this process, teachers can gain a deeper understanding of international students’ cultural backgrounds and individual needs, enabling them to promptly identify potential cultural conflict risks and implement preemptive measures. Additionally, such positive teacher—student relationships contribute to the creation of a harmonious campus environment, providing proactive support for international students’—cultural adaptation.

In conclusion, universities can mitigate misjudgments stemming from cultural ignorance or inadequacies by systematically imparting cross-cultural theories, customs, religious taboos, and value systems to faculty members. Through cross-cultural communication decoding training and workshops aimed at resolving value conflicts, educational institutions can enhance teachers’ proficiency in cross-cultural communication and conflict resolution mediation. Moreover, by organizing in—depth interviews with international students and scenario—based experiential activities, educators can cultivate cultural empathy and foster a favorable cross-cultural environment.

It is of great significance to acknowledge that the post-pandemic era is concurrent with the rapid digital development. In this context, excessive dependence on digital platforms has exacerbated the mental health risks for students. Chinese international students are increasingly relying on social media for information, while their offline social skills have declined and social distancing has become more prominent. This prolongs their cross-cultural adaptation period and may result in a decrease in cultural integration.

To address this issue, universities should organize cultural exchange programs, such as China Day events, cultural immersion tours, and talent showcases. Encouraging peer-to-peer mentorship initiatives will assist students in cultivating cultural awareness in multicultural settings, improving emotional intelligence and behavioral competence in cross-cultural communication, and ultimately attaining successful academic adaptation.

To optimize psychological counseling services, it is advisable to learn from international exemplary practices. In Australia, international students can avail themselves of counseling services via the Australian Living Helpline and depression prevention programs, which have proven to be highly accessible. These approaches provide valuable references. Enhancing service accessibility is of utmost importance by integrating counseling channels into students’ daily lives. Universities should promote these resources through official social media accounts and communication platforms, enabling students to conveniently book mental health consultation appointments. Schools can also publicize dedicated hotlines to guarantee immediate contact. For Southeast Asian universities with a large number of international students, developing a “Digital Cultural Adaptation Platform” would be a prudent choice. This platform could incorporate applications in Southeast Asian languages, integrated features such as Chinese language learning resources, counseling booking systems, and campus life guides, as well as self-assessment tools for anxiety monitoring. High-risk students can receive personalized interventions. Through these strategies, theoretical understandings of cultural adaptation stress and academic anxiety can be translated into localized and targeted support measures. This approach facilitates Southeast Asian students in building psychological resilience, transitioning from passive anxiety management to proactive adaptation.

### 5.1 Enhance teaching facilities and optimize curriculum design

Research suggests that learning environments and academic instruction exert a significant influence on the academic adaptation of international students. To tackle this issue, universities should adopt specific strategies to improve teaching facilities while optimizing course content. Firstly, institutions are required to establish coherent curriculum frameworks with comprehensive planning, and conduct accurate evaluations of both teaching materials and student capabilities. For advanced courses in the later years, foundational content should be introduced during the freshman and sophomore years to gradually increase the difficulty level while reducing academic pressure. Secondly, addressing psychological adjustment and language proficiency disparities is equally important. Language improvement necessitates integrating specialized Chinese language courses into the curriculum, breaking down learning difficulties into manageable parts to relieve academic stress and enhance adaptation. Beyond core courses, elective programs customized for international students should be expanded to meet their requirements, incorporating culturally relevant content based on their preferences. Promoting cross-cultural adaptation through immersive experiences can expedite academic integration. This may involve offering comparative cultural studies courses and integrating cultural elements into professional subjects, enabling students to perceive Chinese cultural characteristics from foreign perspectives. Additionally, educators should adopt participatory and experiential teaching methods, extending classroom interactions to Chinese society. By involving students in practical activities that reflect Chinese cultural values, abstract conceptual barriers can be effectively overcome.

Moreover, academic performance anxiety exerts a significant influence on international students’ adaptation to the Chinese academic environment. Disparities in language proficiency directly impede access to academic support resources, including tutoring sessions and thesis guidance. To tackle this issue, educational institutions ought to implement inclusive assessment criteria and adopt flexible evaluation approaches. For example, Southeast Asian students could gain advantages from a “multidimensional assessment system”: permitting bilingual thesis outlines during the initial writing stage before progressing to full Chinese submissions, and providing culturally relevant topics such as “practical reports” and “cross-cultural comparative studies” to enhance academic confidence through familiarity with the academic field. Institutions should eschew one—dimensional evaluations and reinforce process—oriented assessments to alleviate anxiety stemming from language barriers that may lead to expression distortions or errors, which could potentially impact academic grades. Additionally, academic departments could establish “academic adaptation mentors” who conduct weekly small—group sessions to address specific challenges, such as “critical thinking development” and “literature search techniques.” Qian and Yu (2023) revealed that when international students in China encounter intense academic anxiety, their campus adaptation and psychological resilience decline, while depression levels rise. Higher academic grades are associated with lower anxiety levels. Campus adaptation and academic resilience manifest significant mediating effects, along with a notable chain mediation effect between these two factors.

### 5.2 Efforts will be made to enhance the education of international students and standardize student management

An analysis of the influence of living adaptation on academic adaptation indicates that Southeast Asian international students in China generally encounter varying degrees of adjustment difficulties in terms of climate, transportation, dietary patterns, and service systems. They also exhibit dissatisfaction with dormitory management regulations. Orientation programs play a pivotal role in facilitating their smoother adaptation to local circumstances and university settings. As a crucial transitional stage for international students as they assume their new roles, these programs act as a bridge linking them to Chinese universities. Effective orientation measures can substantially improve students’ academic adaptation by assisting them in integrating into the learning environment and accomplishing their studies successfully.

To cultivate a sense of belonging among international students, universities are required to implement human—centered approaches in student management. Prioritizing the needs of students should serve as the foundation of institutional care.

Prior to enrollment, schools ought to offer comprehensive living guidance via email and telephone communication. Upon the students’ arrival, orientation programs are crucial in enabling newcomers to seamlessly integrate into campus life. These orientation sessions should encompass the utilization of libraries, digital resources, and teaching facilities. Educators are also obligated to elucidate China’s educational framework, learning methodologies, and academic attitudes.

Throughout the entire process, spanning from initial enrollment to graduation procedures, administrative staff assume a significant role. Counselors act as key intermediaries, handling daily affairs while closely monitoring the mental well-being of students, especially during their initial adaptation phase. They organize class activities that promote cultural exchange between Chinese and international students and strengthen the sense of community.

To assist international students in addressing daily life and academic challenges, counselors can arrange for senior students to share their experiences. The guidance provided by upperclassmen not only helps new students establish a stronger connection with the campus environment but also offers practical advice for a more seamless adaptation.

These measures aid students in surmounting adjustment barriers, adapting to campus management, making mental preparations, minimizing cultural shock, and ultimately facilitating their academic success.

### 5.3 Establish student mutual-assistance clubs to offer peer support

The academic adaptability of Southeast Asian undergraduate international students in China is significantly affected by learning competencies, which include elements such as motivation and study methodologies. Internally—driven motivations for overseas study also play a pivotal role in academic adaptation. Strengthening these internal motivations can effectively enhance students’ academic integration. Student organizations serve as essential platforms for enhancing international students’ learning capabilities and reinforcing their intrinsic motivation. Scholars stress the significance of creating social integration opportunities for international students. By organizing cultural exchange activities and language courses, interactions between international and local students can be promoted (Ma et al., 2024; Dost, 2025). The establishment of student support groups enables students to share academic and daily—life experiences while addressing challenges. These groups cultivate a more relaxed atmosphere where students are at ease discussing academic and personal matters. Specifically, specialized academic clubs can hold problem—solving sessions, and regular student organizations, especially those with high international participation, can occasionally organize study exchange events. Through peer-to-peer sharing of adaptation strategies, academic and personal experiences, such collaborative approaches directly fulfill the goals of mutual assistance.

The establishment of student mutual-aid clubs can enhance the social interactions of international students. As social entities, individuals acquire a sense of belonging through social activities and connections. Positive interpersonal relationships can even serve as essential support and motivation for international students during their study in China. Through club activities, international students can interact with more Chinese peers in social settings and improve their communication skills, which is of great significance for their adaptation to Chinese social interactions.

The creation of student mutual-aid clubs also deepens cultural understanding and strengthens study motivation. With the encouragement of relevant policies, Chinese university students currently establish a variety of clubs that play a crucial role in uniting international students and promoting Chinese culture. On one hand, educational institutions could encourage students to form clubs related to Chinese culture, such as the Kung Fu and Tea Art Society or the Pear Garden Opera Club. During the recruitment process, actively attracting foreign students to join these clubs enables them to better experience and understand Chinese culture, thereby enhancing their motivation to learn about China.

Moreover, schools should encourage students to establish cultural clubs that showcase the traditions of their home countries, which facilitates cultural exchanges between Chinese and international students. This approach also contributes to strengthening cultural confidence. When international students perceive that their country’s culture is appreciated and respected by others, they develop a greater sense of recognition and belonging towards the current social norms and events.

### 5.4 Rigorous language level evaluation and emphasis on the enhancement of students’ professional Chinese proficiency

In our investigation of individual influencing factors, it was found that Chinese language proficiency exerts a significant influence on the academic adaptation of international students. Wilczewski and Alon (2023) also indicated that language competence is a critical variable for international students’ adaptation in digital learning settings. Proficiency in a second language (e.g., English) plays a vital role in cross-cultural adaptation, especially during interactions with local students and faculty. Through interviews, it was further revealed that many international students encounter no substantial difficulties in daily Chinese communication. Nevertheless, the more specialized Chinese proficiency necessary for academic adaptation remains the key constraining factor. Therefore, universities should concentrate on initial language proficiency evaluations and subsequent development of professional Chinese skills. This strategy will effectively elevate the academic adaptation levels of Southeast Asian international students in China.

Since the inception of the “Belt and Road” Initiative, a growing number of international students have opted to pursue their studies in China. With the broadening scope of government scholarships and provincial-level grants, language proficiency assessment has emerged as a crucial criterion for university admissions. The HSK Chinese Proficiency Test continues to be the predominant approach for language evaluation in Chinese universities. Generally, applicants for undergraduate and postgraduate programs are required to attain HSK Level 4 and Level 5, respectively. Although most Chinese institutions place significant emphasis on HSK scores when assessing language proficiency, this standardized test mainly evaluates communicative competence rather than specialized academic skills. While such criteria may facilitate students’ adaptation to daily life, they often pose substantial obstacles to academic pursuits due to the discrepancies between students’ language proficiency and the difficulty levels of courses. To comprehensively evaluate language abilities and support professional development, universities should conduct both HSK assessments and subject-specific Chinese proficiency tests during the admission process.

Upon the enrollment of international students in Chinese academic programs, the cultivation of their professional Chinese proficiency ought to be accorded top priority. Distinct from communicative Chinese competence, professional Chinese proficiency is specifically tailored to support specialized learning within academic disciplines, signifying students’ capacity to acquire technical knowledge via the Chinese language. The research conducted by Di (2022) indicates that cultural adaptation showed a moderately significant positive correlation with Chinese language proficiency (*p* < 0.05), and a significantly positive correlation with enrollment management, teaching management, differentiated management, recognition of administrative staff, and teaching satisfaction (all *p* < 0.01). However, no significant relationship was found with age, educational background, or length of stay in China. Consequently, throughout the student development process, educational institutions should devise professional Chinese courses that are in line with disciplinary requirements. This strategy enhances students’ technical language capabilities, promotes effective engagement in courses, and mitigates anxiety associated with academic adjustment.

In summary, international students encounter multi-dimensional academic adaptation challenges in the post-pandemic era. Reinforcing psychological counseling and offering mental health support has become especially significant. Educational institutions, governments, and society are required to cooperate to provide comprehensive support services, which can assist international students in better integrating into new cultural environments and attaining academic and personal development.

## Data Availability

The original contributions presented in the study are included in the article/supplementary material, further inquiries can be directed to the corresponding author.
